# Secondary analysis of retrospective and prospective reports of adverse childhood experiences and mental health in young adulthood: Filtered through recent stressors

**DOI:** 10.1016/j.eclinm.2021.101094

**Published:** 2021-08-19

**Authors:** Sara N. Naicker, Shane A. Norris, Linda M. Richter

**Affiliations:** a DSI-NRF Centre of Excellence in Human Development, University of the Witwatersrand, Johannesburg, South Africa; b SAMRC/Wits Developmental Pathways for Health Research Unit, Faculty of Health Sciences, University of the Witwatersrand, Johannesburg, South Africa

**Keywords:** Adverse childhood experiences, ACES, Retrospective, Prospective, Psychological distress, Mental health

## Abstract

**Background:**

Evidence has identified the detrimental effects that adverse childhood experiences (ACEs) have on outcomes across the life course. We assess associations between prospective and retrospective ACEs and mental health in young adulthood and the influence of recent stressors.

**Methods:**

Secondary analysis of a sample of 1592 young adults from the Birth to Twenty Plus cohort, from 1990 to 2013, were assessed throughout their first 18 years for prospective ACEs. Retrospective ACEs and an assessment of mental health were collected at the 22–23-year data point.

**Findings:**

Prospective physical and sexual abuse are associated with an increased risk of depression (OR 1·7 [95% CI 1·37–1·93, *p* = 0·034], and OR 1·8 [95% CI 1·27–2·07, *p* = 0·018], respectively). Retrospective emotional abuse/neglect is associated with increased anxiety (OR 1·8 [95% CI 1·32–2·36, *p* = 0·000]), depression (OR 1·6 [95% CI 1·08–2·25, *p* = 0·018]) and overall psychological distress (OR 1·6 [95% CI 1·18–2·17, *p* = 0·002]). Prospectively reporting four or more ACEs is associated with a twofold increase in risk for overall psychological distress (OR 2·2 [95% CI 1·58–3.12, *p* = 0·008]). Retrospectively reporting four or more ACEs is associated with increased likelihood of somatization (*p* = 0·004), anxiety (*p* = 0·002), depression (*p* = 0·021), and overall psychological distress (*p* = 0·005).

**Interpretation:**

Both individual and combined retrospective and prospective ACEs are related to mental health in young adulthood. Recent stressors reinforce this relationship; the likelihood of those who report more ACEs experiencing psychological distress increases when adjusting for recent stressors.

**Funding:**

Wellcome Trust (UK), South African Medical Research Council, Human Sciences Research Council, University of the Witwatersrand and supported by the DSI-NRF Centre of Excellence in Human Development.

**Research in Context**

**Evidence before this study**

Experiences of adversity in childhood have been linked to increased risk of poor mental health outcomes in later life, with less evidence in the young adult population. Results from previous analyses show the prevalence of reported adverse childhood experiences (ACEs) vary when assessed prospectively and retrospectively in the same sample, however the evidence in LMICs is scarce. Adult stress, independent of early adversity, has been linked to mental health problems, but is also hypothesized to act in conjunction with histories of adversity to either sensitize individuals to future stressors, disrupt coping strategies or aggravate negative mental health outcomes.

**Added value of this study**

The findings of this study indicate that the timing, type and number of reported ACEs work together to impact on mental health outcomes in young adulthood. Both prospective and retrospective reporting of four or more ACEs are associated with poor mental health, even though the prevalence of retrospective reports of individual ACEs decreases over time. When adjusted for the number of recent stressors, the likelihood of overall psychological distress increases as the number of ACEs increases.

**Implication of all the available evidence**

While the concept of adverse childhood experiences constitutes a range of negative exposures for children (1) some are more deleterious on mental health in young adulthood, independent of others, (2) the cumulative risk of ACEs should not be ignored since poor mental health in young adulthood is also associated with the total number of ACEs reported, and (3) females report exposure to a similar number of adverse childhood experiences prospectively and fewer retrospectively than their male counterparts but are at twice the risk of poor mental health.

Alt-text: Unlabelled box

## 1 Introduction

A large body of research documents associations between adverse childhood experiences (ACEs) and health and well-being [1]. Risk factors for chronic disease including overweight and obesity [2,3], and smoking [4,5] have been linked to ACEs. The Kaiser ACE study found that as the number of exposures to ACEs increased, so did the prevalence and risk of, amongst others, alcoholism, use of illicit drugs, risky sexual behavior and having a history of a sexually transmitted infection [6]. Growing evidence suggests that ACEs are inter-related; [7] for example, childhood sexual abuse often occurs in the presence of other ACEs [8], emphasizing the need for ACEs to be assessed comprehensively.

Environmental, socioeconomic and behavioral exposures, whether independent or clustered together, can be compounded over time to manifest in an accumulation of risk, which can affect adult health and wellbeing either through cumulative damage over time or by the biological embedding of adversities during sensitive developmental periods [9,10]. The value of the ACE score, the total number of ACEs to which an individual reports having been exposed, lies in the ability to examine the cumulative impacts of ACEs on later life outcomes. The evidence describes the relationship between the extent of ACEs and social and health problems as one that predicts the risk to increase in a strong and graded manner as the number and severity of ACEs increase. Growing evidence shows that some ACEs – and some combinations ‒ have a more deleterious effect on health and well-being than others [11], and may possibly have differential effects on different outcomes. For example, one study linked a cluster of ACEs related to abuse and neglect to higher severity bipolar disorder and schizophrenia, compared to a cluster of social support-related ACEs [12]. In another study on the clustering of individual ACEs, a child maltreatment and peer victimization group was associated with double the odds of self-rated poor physical health and three times the odds of self-rated poor mental health, compared to a household challenges grouping which was linked to an almost 3-fold chance of reporting poor physical health and six-times odds of reporting poor mental health [13].

In addition to the clustering and relative weight of certain ACEs, the method of data collection – essentially the timing of reporting – has been explored. Studies have found low to moderate agreement between prospective and retrospective ACEs, depending on the type of ACE [14,15], and have also found differential associations with outcomes. One such study found that while prospective and retrospective reports showed associations with outcomes in midlife, retrospectively reported ACEs showed stronger associations with outcomes that were subjectively assessed compared to those objectively assessed [15]. Further complexity abounds when the nature of prospective reports are examined; while these are taken to be near-contemporaneous accounts of events, prospective data is often still recalled or reported by participants, or in the case of young children – caregivers. Prospective accounts such as court records of abuse or substantiated cases of adversity are considered objective but are challenging to integrate into research for a number of reasons; they also represent only a proportion of real cases – those considered serious enough to go through the child protection system, and those that are actually reported [16]. However, emerging evidence points to the association of retrospective reports with negative outcomes, including psychopathology, even when there are inconsistencies with objective evidence of adversity [15,17]. Moreover, recent life stress has been hypothesized to play some role in the relationship between early adversity and adult outcomes. Theories put forward include the dysregulation of stress response systems which result in maladaptive responses to subsequent stress or the increase in risk for future adversity based on exposure to adversity in childhood [18,19]. Findings from the Birth to Twenty Plus cohort show that high levels of adult stress significantly increased the likelihood of psychological distress for those with high levels of ACEs, and posit a possible mediation effect between ACEs and psychological distress [20]. A number of studies have linked ACEs to poor mental health in adulthood [21], [22], [23], [24], [25]. The long-term effects of ACEs on mental health during the early adult years in low-middle-income settings have been understudied and where they have, most studies include clinical or cross-sectional samples [26]. Studies that have been conducted confirm the relationship between exposure to ACEs and poor adult mental health [27]. The aim of this study is to assess the associations of prospectively and retrospectively reported accounts of ACEs to the mental health of a young adult sample from a peri-urban, historically disadvantaged South African context. In addition, this study will explore potential mediating or moderating effects of recent stressors on the relationship between ACEs and mental health outcomes. The study will contribute to the ACEs literature by exploring how different approaches to ACEs measurement – single, cumulative and prospective versus retrospective, can be associated with mental health outcomes in a young adult sample. Clarity on the timing, type and number of ACEs that are linked to persistent negative outcomes is critical for the development of appropriate interventions, particularly in contexts like South Africa where there exists a large gap between the burden of mental health problems and the resources available to address it [28].

## 2 Methods

### 2.1 Study design and participants

The Birth to Twenty Plus study (BT20+) is a South African birth cohort of 3273 singleton children born to mothers who were residents of Soweto-Johannesburg in a 7-week period of enrolment in 1990. The study is unique in that it is the largest and longest running study of child and adolescent health and development in Africa. Current participants are 30 years old, have been assessed up to 22 times and, since 2005 when the first participant birth occurred, includes the 3rd generation of the cohort. A detailed description of the study, its birth cohort and participants is published elsewhere [29]. This study uses data from birth to age 22–23-years old for prospective and retrospective reports of ACEs and covers the period between 1990 and 2013. A total of 1636 participants were surveyed at the 22–23-year wave and a sample of 1592 participants from this group that had both retrospective and prospective reports of ACEs was included in this analysis. Ethical clearance was obtained from the Witwatersrand University Committee for Research on Human Subjects (protocol number: M140726). All participants and/or their caregivers gave informed written consent for the data reported.

### 2.2 Procedures

#### 2.2.1 Adverse childhood experiences and recent stressors

Adverse childhood experiences have been defined as physical abuse, sexual abuse, emotional abuse and/or neglect, child separation, divorce or parent separation, parent death, exposure to violence, exposure to intimate partner violence (IPV), chronic unemployment, household substance abuse, household legal trouble, household serious illness or disability, and household death. The ACEs survey questions are included in Supplementary Table A. For prospective reports, caregivers were asked to report on their children at participant ages 5, 7 and 11, and participants provided self-reports at ages 11, 15 and 18. A participant was recorded as having experienced a particular ACE if there was a positive response at any one of these time points. For the retrospective report, participants were asked at the 22–23-year wave to indicate if they had experienced each of the ACEs during the first 18 years of their life. A full detailed account of individual ACEs reported at each of the 7 time points, as well as an analysis of the level of agreement between sources and timing, has been published [14]. In summary, that analysis found the reports of prospective and retrospective ACEs, used in this study, had little overall agreement; 80% of the kappa values were below the moderate agreement cut-off of *k* = 0·41. The highest levels of agreement were between reports on parental death (*k* = 0·52) and household death (*k* *=* *0*·51). Reporting on early life ACEs by caregivers (at ages 5, 7 and 11) showed the greatest concordance with retrospective reports of ACEs on sexual abuse (91·0% agreement), physical abuse (87·7% agreement), and exposure to intimate partner violence (80·2% agreement) [14].

For the purposes of this paper, we conceptualise the ACEs directly impacting an individual – physical, sexual, and emotional abuse – as proximal ACEs, and those occurring in their environment – exposure to IPV, household illness, chronic unemployment – as distal ACEs. For ease of reading, retrospectively reported ACEs may be referred to as ‘retrospective ACEs’ and vice versa; similarly, for individual ACEs we may use the shorthand ‘prospective physical abuse’ rather than ‘prospectively reported physical abuse’ but the method of data collection for all ACEs is either self-reported or parent-reported (in the case of children under the age of 7 years).

An assessment of recent stressors, adapted from the Township Life Event Scale [30], was added to the analysis. Participants were asked at the 22–23-year data collection wave to indicate if they had experienced any of 9 negative life events ‒ considered stressors ‒ in the past 6 months. The 9 events included violence in the household (1), workplace (2) or community (3), household illness (4), disability (5) or death (6) in the family, household substance abuse (7), alienation from family (8), and legal trouble (9). Full questions are available in Supplementary Table A.

### 2.3 Mental health outcomes

Young adult mental health was assessed using the self-reported GHQ-28 which comprises 4 sub-scales of 7 items each probing for somatic symptoms, anxiety and insomnia, social dysfunction, and major depression. The 28 items are scored in a binary 0011 method. Higher scores on the GHQ-28 represent higher levels of psychological distress. The GHQ-28 is used in epidemiological studies as a screening for minor psychiatric morbidity caseness (clinically significant anxiety and/or depression). Any score above 4 on a subscale and above 23 on the total scale indicates the presence of distress or a positive diagnostic [31].

### 2.4 Statistical analysis

Data was analyzed using STATA statistical software version 13·0. The ACEs data was transformed into a retrospective and a prospective categorical score for each participant as follows: 0= ‘no reported ACEs’, 1= ‘one reported ACE’, 2= ‘two reported ACEs’, 3= ‘three reported ACEs’ and 4= ‘four or more reported ACEs’. In parts of the analyses outcomes are compared between ‘less than four’ and ‘four or more’ reported ACEs. There are currently no guidelines on the ACEs scoring in the available literature but some studies do point to the ‘four or more’ cut-off functioning as a threshold level, with noticeable deviations in a range of outcomes at that mark [6,32].

The four mental health outcomes, somatization, anxiety, social dysfunction, and depression were transformed into categorical data and the co-occurrence of psychological distress with reports of ACEs was evaluated using the chi-square statistic. Unadjusted effects of each individual ACE, followed by each composite measure of ACEs, separately for prospective and retrospective ACEs, were tested for effects on somatization, anxiety, social dysfunction, depression and GHQ total. Five adjusted logistic regression models were fitted including significant predictors from the unadjusted models, controlling for sex, socio-economic status, maternal education and recent stressors, to estimate the association between the ACE scores and each outcome. Odds ratios and 95% confidence intervals were calculated separately for each outcome. In the fully adjusted models, retrospective and prospective ACEs are entered in the same model together with the selected covariates, therefore the ORs for prospective ACEs indicate the contribution of prospective ACEs independently from retrospective ACEs and vice versa. Factorial analysis of variance was used to test for the unique contribution of prospective and retrospective reports of ACEs, as well as any interactions between them, to the variance in each mental health outcome.

Regression analysis was used to test for mediation and moderation effects of recent stressors on mental health outcomes. Factorial analysis of variance tested for interaction effects between retrospectively and prospectively reported ACEs and recent stressors on psychological distress. These analyses yielded no significant results and the recent stressors were subsequently added to the regression models as a covariate.

### 2.5 Role of the funding source

The funder of the study had no role in the study design, data collection, data analysis, and data interpretation, or writing of the report. All authors had full access to the data and accept final responsibility to submit for publication. Access to the data is available to all authors for as long as they are part of the study team. Authors are permitted to keep their own copy of a dataset specific to a publication ad infinitum.

## 3 Results

### 3.1 Sample and data description

Of the initial 3273 participants recruited in 1990, 1636 were surveyed at the 22–23-year wave in 2017, representing a loss to follow-up of 50%. ACEs data was available for 1592 of the 1636 participants surveyed in 2013, the remaining 42 participants were not included in this analysis. A description of sample demographics at recruitment and at the 22–23-year wave, by sex, is shown in Table 1.

**Table 1:** Demographic profile of sample at baseline and at 22–23-year wave, by sex.

Timepoint	Baseline^a^			22–23-year wave^b^		
	Total N (%)	Male N (%)	Female N (%)	Total N (%)	Male N (%)	Female N (%)
Marital status						
Married or cohabitating	558 (35·3)	282 (37·1)	276 (33·6)	786 (50·2)	340 (45·2)	446 (54·9)
Single or separated	1024 (64·7)	479 (62·9)	545 (66·4)	779 (49·8)	412 (54·8)	367 (45·1)
Education						
No formal education	193 (13·1)	97 (13·8)	96 (12·5)	0 (0)	0 (0)	0 (0)
Primary school	687 (46·5)	337 (47·8)	350 (45·4)	3 (0·2)	1 (0·2)	2 (0·3)
Secondary school	492 (33·3)	221 (31·4)	271 (35·2)	942 (70·0)	398 (69·6)	544 (70·4)
Post-school education	104 (7·1)	50 (7·1)	54 (7·0)	400 (29·7)	173 (30·2)	227 (29·4)
Socioeconomic status^a^						
Quintile 1	225 (15·4)	114 (16·2)	111 (14·5)	505 (32·8)	250 (33·7)	255 (31·8)
Quintile 2	269 (18·4)	130 (18·5)	271 (35·5)	242 (16·7)	117 (15·8)	125 (15·6)
Quintile 3	511 (34·9)	240 (34·2)	271 (35·5)	309 (20·0)	162 (21·9)	147 (18·4)
Quintile 4	297 (20·3)	148 (21·1)	149 (19·5)	272 (17·6)	118 (15·9)	154 (19·2)
Quintile 5	164 (11·2)	70 (10·0)	94 (12·3)	214 (13·9)	94 (12·7)	120 (15·0)
Adverse childhood experiences						
0	294 (23·1)	137 (22·9)	157 (23·2)	139 (8·7)	61 (43·9)	78 (56·1)
1	469 (36·8)	214 (35·8)	255 (37·7)	277 (17·4)	132 (47·7)	145 (52·3)
2	188 (14·7)	95 (15·9)	93 (13·7)	293 (18·4)	113 (38·6)	179 (61·4)
3	122 (9·6)	58 (9·7)	64 (9·5)	279 (17·6)	138 (49·5)	140 (50·5)
≥4	202 (15·8)	94 (15·7)	108 (16·0)	604 (38·0)	320 (53·0)	283 (47·0)

a Measure of maternal or household characteristics.

b Measure of the participant's characteristics.

ACE scores were computed for each participant who had data for at least 10 of the 13 ACEs; those with fewer than 10 – or 3 or more missing data points – were excluded from the analytic dataset. For prospective data, missing data was imputed from previous and subsequent waves of data to compose comprehensive accounts. In the retrospective data, all variables except for parental divorce (17%) had less than 10% missing values. The analysis was restricted to cases with data on the exposures, only cases with data for both prospective and retrospective ACEs were included. Missing data ranged from 1·07% to 3·52% on outcome variables, and between 0·19% and 7·91% on the covariates (Supplementary Table B). The distribution of ACEs among cases with data was not substantially different from those cases without data on a specific variable, and significant differences are due to the small number of missing cases (Supplementary Table C). The largest proportions of missing data were among covariates (up to 7·91%) and these cases were dropped through listwise deletion during the regression analyses. Given the relatively small proportions of missing data, and the comprehensiveness of ACE data, no further handling of missing data was done and all analyses assumes data are missing at random, which the authors concede is a limitation.

### 3.2 Prevalence of ACEs

Table 2 shows the frequency of ACEs by source of report and sex. Exposure to individual ACEs is summed to create an ACE score. Changes in the prevalence of ACEs by report (prospective versus retrospective) and source (self- versus parent-) in this cohort has been explored in greater detail in a previous publication [14] and are summarized in the methods section. The proportion of participants who report experiencing four or more ACEs drops by more than half, from 87·4% to 38·0%, when reported retrospectively compared with prospective reporting. The prevalence of all ACEs decreases from prospective to retrospective reporting, with the exception of reports of parental death which increases from 22·4% to 27·9%. The greatest decreases are in reports of physical and sexual abuse (87% and 90% decrease, respectively), exposure to violence inside and outside of the household (73% and 61% decrease, respectively), and chronic unemployment in the household (49% decrease). Reports of emotional abuse/neglect are much the same regardless of when it is reported, with 36·2% prospectively and 34·7% retrospectively. Overall, individuals tend to report fewer ACEs retrospectively than they do prospectively, with 12·5% of participants reporting fewer than four ACEs prospectively compared to 62·0% retrospectively.

**Table 2:** Prevalence of ACEs by report type and sex.

Source of report	Prospective report			Retrospective report		
	Total N (%)	Male N (%)	Female N (%)	Total N (%)	Male N (%)	Female N (%)
Adverse childhood experiences						
Physical abuse	880 (55·3)	457 (51·9)	423 (48·1)	118 (7·4)	67 (56·8)	51 (43·2)
Sexual abuse	613 (38·5)	296 (48·3)	317 (51·7)	63 (4·0)	22 (34·9)	40 (65·1)
Emotional abuse/neglect	577 (36·2)	282 (48·9)	294 (51·1)	552 (34·7)	278 (50·4)	272 (49·6)
Child separation	244 (15·3)	105 (43·0)	139 (57·0)	–	–	–
Divorce/separation	816 (51·3)	382 (46·8)	434 (53·2)	593 (37·3)	283 (47·7)	309 (52·3)
Parental death	357 (22·4)	182 (51·0)	173 (49·0)	443 (27·9)	230 (51·9)	212 (48·1)
Exposure to violence	1114 (70·0)	591 (53·0)	522 (47·0)	431 (27·1)	246 (57·1)*	184 (42·9)*
Exposure to IPV	750 (47·1)	422 (56·3)*	326 (43·7)*	202 (12·7)	93 (46·0)	108 (54·0)
Chronic unemployment	1346 (84·5)	645 (47·9)	701 (52·1)	684 (43·0)	346 (50·6)	337 (49·4)
Household substance abuse	740 (46·5)	346 (46·8)	394 (53·2)	438 (27·6)	235 (53·7)	202 (46·3)
Household legal trouble	579 (36·4)	325 (56·1)*	254 (43·9)*	364 (22·9)	202 (55·5)	162 (44·5)
Household illness/disability	984 (61·8)	475 (48·3)	509 (51·7)	553 (34·8)	258 (46·7)	294 (53·3)
Household death	971 (61·0)	458 (47·2)	510 (52·8)	396 (24·9)	193 (48·7)	200 (51·3)
ACE score						
0	8 (0·5)	3 (37·5)	5 (62·5)	139 (8·7)	61 (43·9)	78 (56·1)
1	26 (1·6)	12 (46·2)	14 (53·8)	277 (17·4)	132 (47·7)	145 (52·3)
2	46 (2·9)	13 (28·3)*	31 (71·7)*	293 (18·4)	113 (38·6)*	179 (61·4)*
3	120 (7·5)	55 (45·8)	65 (54·2)	279 (17·6)	138 (49·5)	140 (50·5)
≥4	1392 (87·4)	681 (48·9)	710 (51·1)	604 (38·0)	320 (53·0)	283 (47·0)
Total	1592 (100·0)	764 (48)	825 (52)	1589 (100·0)	764 (48·1)	825 (51·9)

*p<·001 – significant differences between males and females.

Categories for ACEs found in the literature were compared across socio-demographic variables (Table 3). Significant differences are indicated by asterisk. In both prospective and retrospective reporting of ACEs, a significantly higher proportion of children living in households with single or separated parents report four or more ACEs (*p* *=* 0·14*)*. Similarly, higher maternal education appears to be associated with fewer reported ACEs both retrospectively and prospectively (*p* = 0·006). Males and females tend to report similar numbers of ACEs prospectively. However, the number of males who report four or more ACEs retrospectively is significantly higher (*p* *=* 0·001*)*. More participants in higher socio-economic quintiles report fewer ACEs both retrospectively and prospectively.

**Table 3:** ACE profiles by demographic variables.

	None N (%)	One N (%)	Two N (%)	Three N (%)	Four or more N (%)	Total N (%)
Prospective Report Total	8 (0·5)	26 (1·6)	46 (2·9)	120 (7·5)	1392 (87·4)	1592 (100·0)
Maternal marital status						
Married or cohabitating	5 (71·4)	10 (40·0)	19 (43·2)	55 (45·8)	469 (33·8)*	558 (35·3)
Single or separated	2 (28·6)	15 (60·0)	25 (56·8)	65 (54·2)	917 (66·2)*	1024 (64·7)
Maternal education						
No formal education	0 (0·0)	2 (8·0)	3 (7·5)	16 (14·4)	172 (13·3)	193 (13·1)
Primary school	0 (0·0)*	5 (20·0)*	19 (47·5)	52 (46·8)	611 (46·5)	687 (46·5)
Secondary school	5 (71·4)*	16 (64·0)*	14 (35·0)	32 (28·8)	425 (32·9)	492 (33·3)
Post-school education	2 (28·6)*	2 (8·0)	4 (10·0)	11 (9·9)	85 (6·6)	104 (7·1)
Sex						
Male	3 (37·5)	12 (46·2)	13 (29·5)*	55 (45·8)	681 (49·0)	764 (48·1)
Female	5 (62·5)	14 (53·8)	31 (70·5)*	65 (54·2)	710 (51·0)	825 (51·9)
Socioeconomic statusa						
Quintile 1	0 (0·0)	4 (18·2)	6 (15·4)	20 (17·5)	192 (15·2)	225 (15·45)
Quintile 2	2 (28·6)	5 (22·7)	5 (12·8)	14 (12·3)	243 (18·9)	269 (18·4)
Quintile 3	1 (14·3)	3 (13·6)	17 (43·6)	38 (33·3)	452 (35·2)	511 (34·9)
Quintile 4	3 (42·9)	6 (27·3)	5 (12·8)	22 (19·3)	261 (20·3)	297 (20·3)
Quintile 5	1 (14·3)	4 (18·2)	6 (15·4)	20 (17·5)*	133 (10·4)*	164 (11·2)
						
Retrospective Report Total	139 (8·7)	277 (17·4)	293 (18·4)	279 (17·5)	604 (37·9)	1592 (100·0)
Maternal marital status						
Married or cohabitating	59 (43·1)	116 (41·9)*	100 (34·6)	95 (34·2)	188 (31·3)*	558 (35·3)
Single or separated	78 (56·9)	161 (58·1)	189 (65·4)	183 (65·8)	413 (68·7^*)^	1024 (64·7)
Maternal education						
No formal education	15 (11·8)	37 (14·2)	29 (10·6)	385 (14·6)	74 (13·3)	193 (13·1)
Primary school	42 (33·1)*	111 (42·7)	127 (46·5)	118 (45·4)	289 (52·0)*	687 (46·5)
Secondary school	55 (43·3)*	90 (34·6)	103 (37·7)	86 (33·1)	158 (28·4)*	492 (33·3)
Post-school education	15 (11·8)*	22 (8·5)	14 (5·1)	18 (6·9)	35 (6·3)	104 (7·1)
Sex						
Male	61 (43·9)	132 (47·7)	113 (38·7)*	138 (49·6)	320 (53·1)*	764 (48·1)
Female	78 (56·1)	145 (52·3)	179 (61·3)*	140 (50·4)	283 (46·9)*	825 (51·9)
Socioeconomic statusa						
Quintile 1	14 (11·1)	37 (14·3)	31 (11·3)	41 (15·9)	102 (18·6)*	225 (15·4)
Quintile 2	23 (18·3)	39 (15·1)	53 (19·3)	48 (18·6)	106 (19·3)	269 (18·4)
Quintile 3	39 (31·0)	93 (36·0)	95 (24·5)	89 (34·5)	195 (35·5)	511 (34·96)
Quintile 4	32 (25·4)	56 (21·7)	62 (22·5)	43 (16·7)	104 (18·9)]	297 (20·3)
Quintile 5	18 (14·3)	33 (12·8)	34 (12·4)	37 (14·3)	42 (7·7)*	164 (11·2)

^a^in quintiles with increasing SES.

*p<·05 – significant differences between ACE scores.

### 3.3 Associations between ACEs and mental health outcomes

Table 4 shows the distribution of mental health outcomes for ACEs reported prospectively and retrospectively, categorized as ‘less than four’ or ‘four or more’. Given patterns in the literature associating adversity in childhood with poor mental health [12,13], it is expected that a greater proportion of respondents who report four or more ACEs will present with psychological distress. Using prospective reports of ACEs there appears to be little significance between reported ACEs and psychological distress (somatization, *p* = 0·465; anxiety, *p* = 0·263; social dysfunction, *p* = 0·522; depression, *p* = 0·050; GHQ total, *p* = 0·273). Using the retrospective reports, there are significant differences in the expression of psychological distress between respondents who report less than four or four or more ACEs on all mental health outcomes (*p* = 0·000), apart from social dysfunction (*p* = 0·360).

**Table 4:** Distribution of mental health symptoms for reported ACES.

Mental health outcome	Prospective report (%)			Retrospective report (%)		
	Less than 4	Four or more	Sig·	Less than 4	Four or more	Sig·
Somatization Below cut-off Above cut-off	125 (63·8) 71 (36·2)	887 (64·4) 491 (35·6)	·465	658 (67·7) 314 (32·3)	354 (58·8) 248 (41·2)	·000
Anxiety Below cut-off Above cut-off	113 (57·7) 83 (42·3)	831 (60·3) 547 (39·7)	·263	650 (66·9) 322 (33·1)	294 (48·8) 308 (51·2)	·000
Social dysfunction Below cut-off Above cut-off	82 (41·8) 114 (58·2)	576 (41·8) 803 (58·2)	·522	410 (42·2) 562 (57·8)	248 (41·1) 355 (58·9)	·360
Depression Below cut-off Above cut-off	174 (88·8) 22 (11·2)	1160 (84·2) 218 (15·8)	·055	865 (89·0) 107 (11·0)	469 (77·9) 133 (22·1)	·000
GHQ Total Below cut-off Above cut-off	145 (75·5) 47 (24·5)	983 (73·1) 361 (26·9)	·273	755 (78·8) 203 (21·2)	373 (64·5) 205 (35·5)	·000

Adding both reports into one model per outcome, we explore the relative contribution of prospective and retrospective reports of ACEs to the variance in each outcome (Supplementary Table D). Significant models were found for somatization [*F*(3, 1570) = 7·18, *p* *=* 0·000], anxiety [*F*(3, 1570) = 25·31, *p* *=* 0·000], depression [*F*(3, 1570) = 26·13, *p* *=* 0·000], and total GHQ [*F*(3, 1532) = 20·73, *p* *=* 0·000], but not for social dysfunction [*F*(3, 1571) = 0·45, *p* *=* 0·7160]. For somatization, only retrospective ACEs have a significant main effect (*p* *=* 0·000) with no interaction effect. Similarly, for anxiety and total GHQ, retrospective ACEs are the only main effect (*p* *=* 0·000) with no interaction effect. There are no significant main or interaction effects for social dysfunction (*p* = 0·518 for prospective ACEs, *p* = 0·503 for retrospective ACEs, and *p* = 0·736 for their interaction). For depression, both prospective and retrospective ACEs have significant main effects at the *p* = 0·000 level, slightly stronger for retrospective ACEs (eta^2^=0·0266) compared to prospective ACEs (eta^2^=0·0122), and a significant interaction effect (*p=*0·0440). Overall, the results indicate that composite measures of retrospective ACEs are associated with somatization, anxiety and the total GHQ; while both prospective and retrospective ACEs contribute to depression. In addition, reporting above the mean (2·5) prospective ACEs and more than four retrospective ACEs is associated with increased scores on depression.

**Fig. 1:**
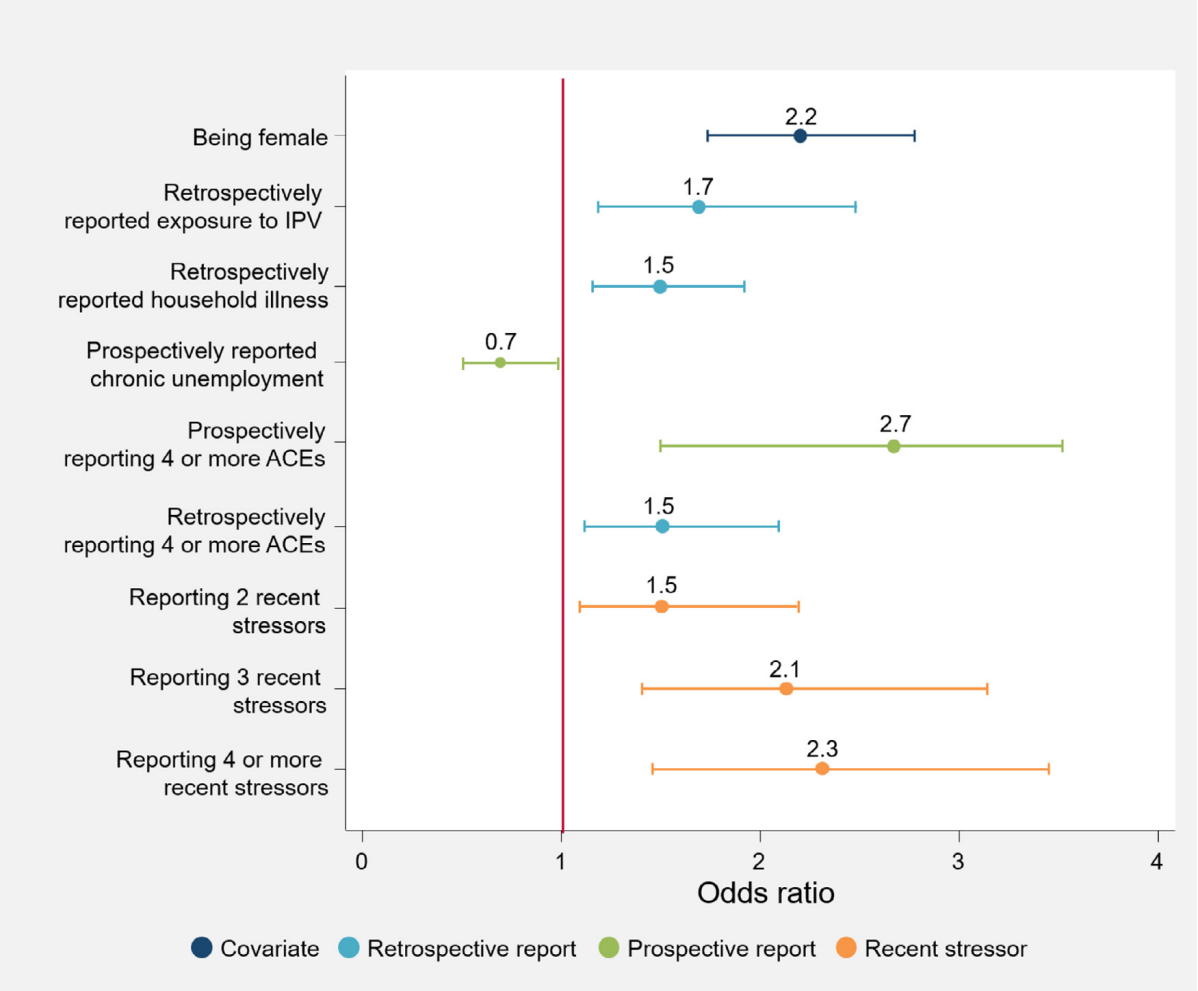
Adjusted effects of reported ACEs and covariates on somatization This figure shows the fold-increase in the odds of experiencing levels of somatization indicating psychological distress given reports of retrospective and prospective ACEs and additional covariates, including recent stressors. Odds ratios (ORs) and their respective confidence intervals for the adjusted model are available in Supplementary Table F.

**Fig. 2:**
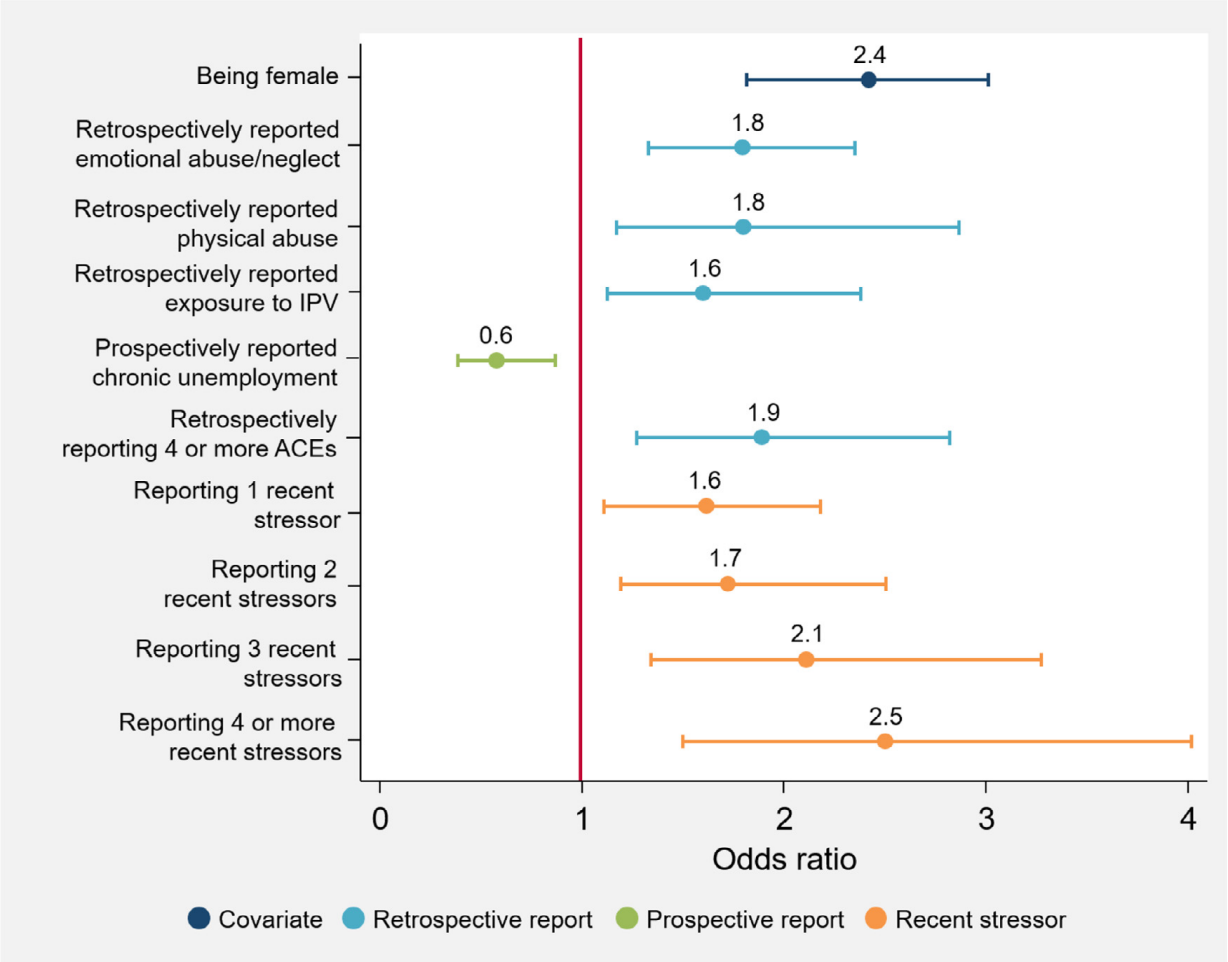
Adjusted effects of reported ACEs and covariates on anxiety This figure shows the fold-increase in the odds of experiencing levels of anxiety indicating psychological distress given reports of retrospective and prospective ACEs and additional covariates, including recent stressors. Odds ratios (ORs) and their respective confidence intervals for the adjusted model are available in Supplementary Table F.

**Fig. 3:**
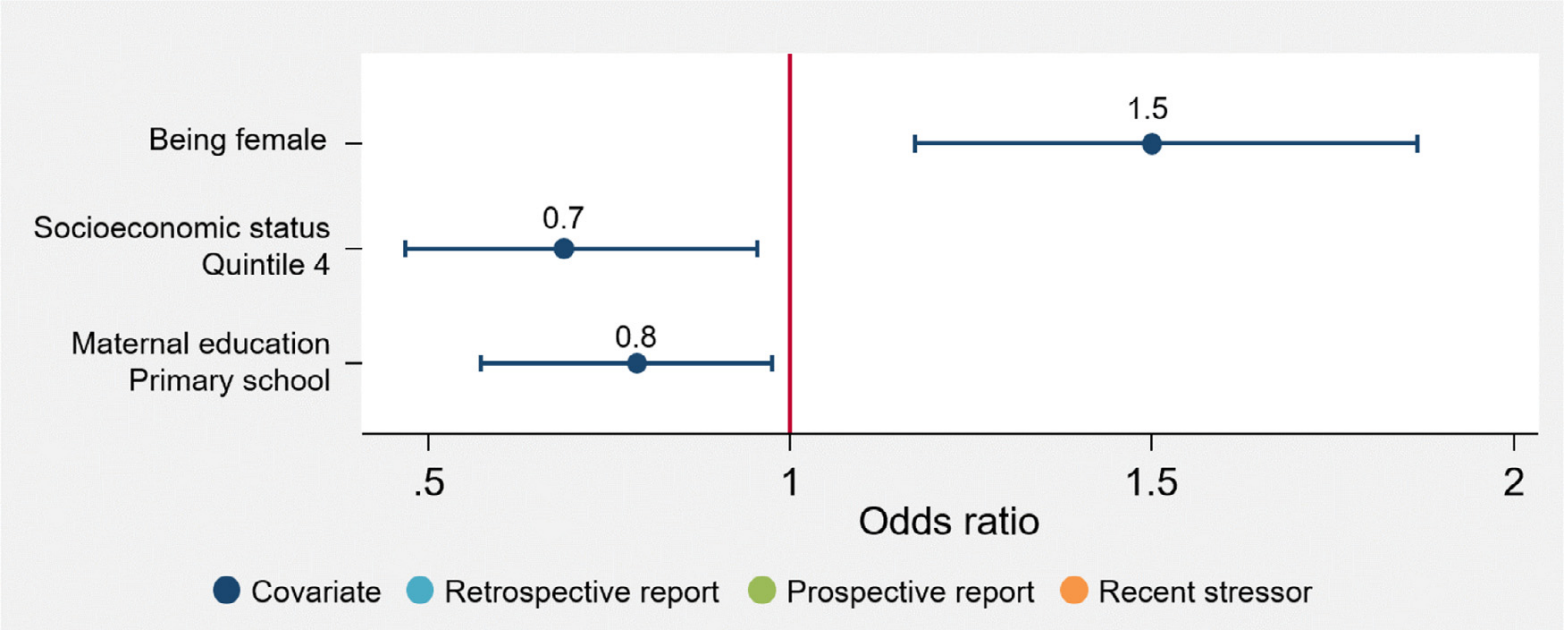
Adjusted effects of reported ACEs and covariates on social dysfunction This figure shows the fold-increase in the odds of experiencing levels of social dysfunction indicating psychological distress given reports of retrospective and prospective ACEs and additional covariates, including recent stressors. Odds ratios (ORs) and their respective confidence intervals for the adjusted model are available in Supplementary Table F.

**Fig. 4:**
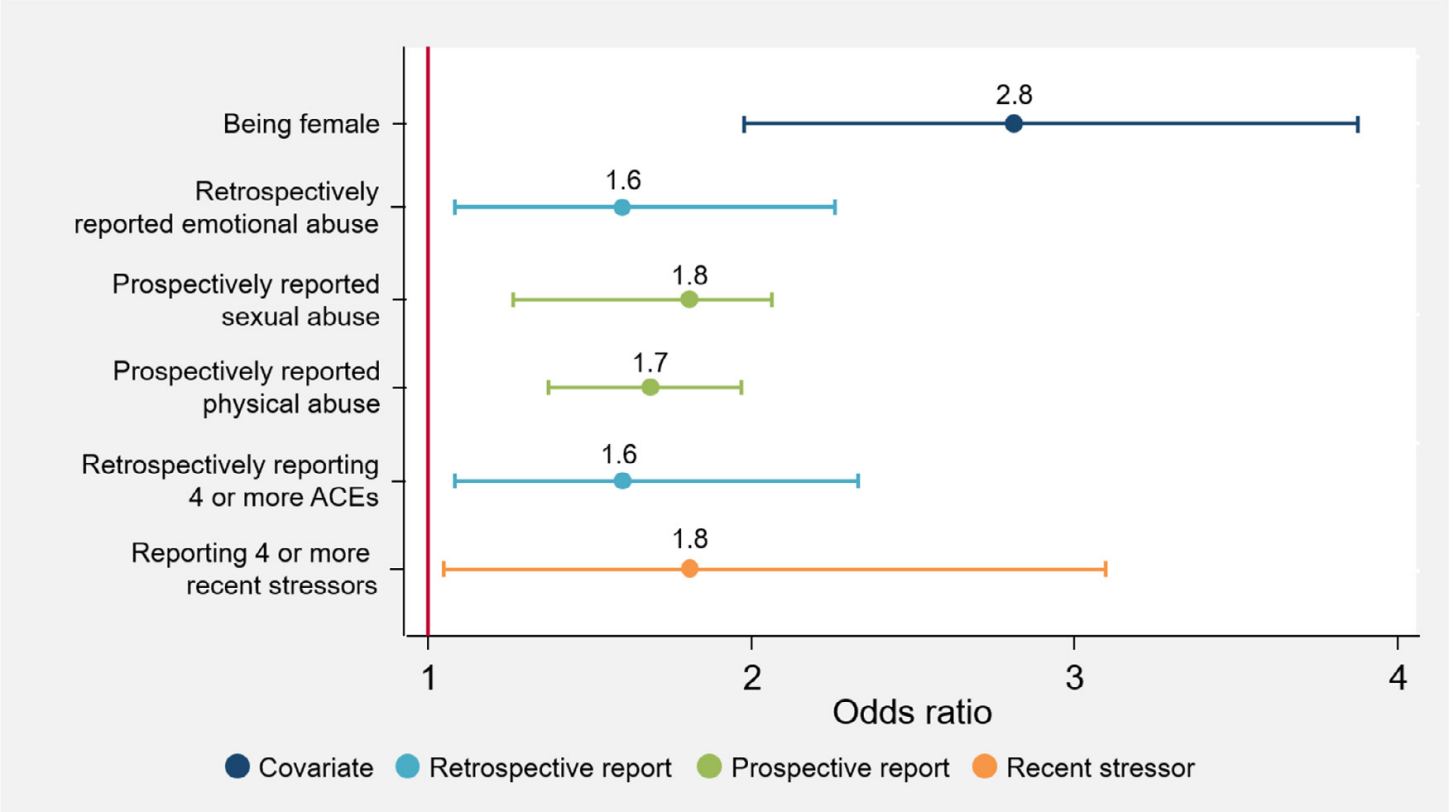
Adjusted effects of reported ACEs and covariates on depression This figure shows the fold-increase in the odds of experiencing levels of depression indicating psychological distress given reports of retrospective and prospective ACEs and additional covariates, including recent stressors. Odds ratios (ORs) and their respective confidence intervals for the adjusted model are available in Supplementary Table F.

**Fig. 5:**
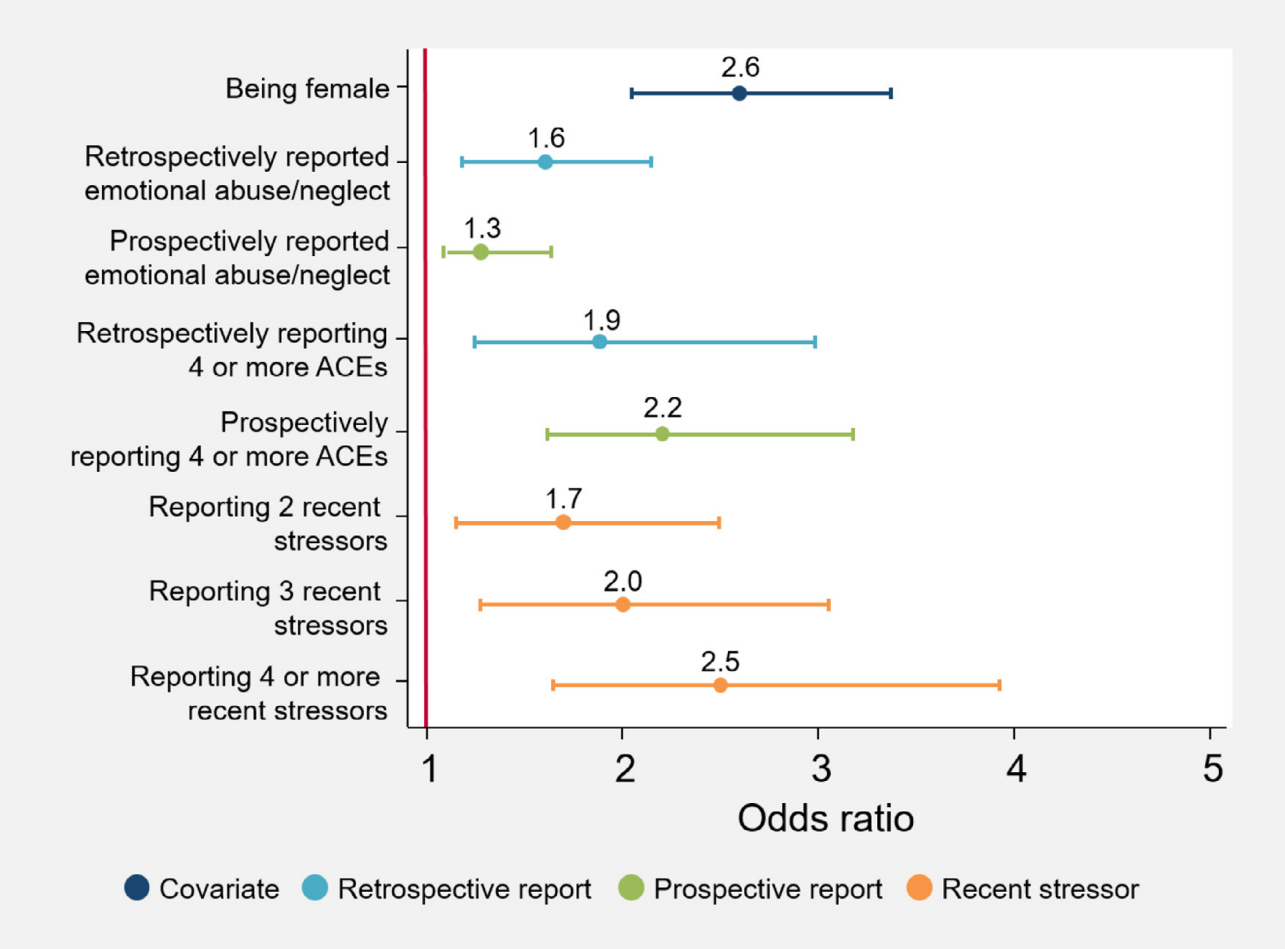
Adjusted effects of reported ACEs and covariates on the total GHQ score This figure shows the fold-increase in the odds of experiencing psychological distress as indicated by total GHQ scores given reports of retrospective and prospective ACEs and additional covariates, including recent stressors. Odds ratios (ORs) and their respective confidence intervals for the adjusted model are available in Supplementary Table F.

Fig. 1, Fig. 2, Fig. 3, Fig. 4, Fig. 5 illustrate the odds ratios for each adjusted model. Results from the unadjusted and adjusted stepwise regressions are included in the supplement as Supplementary Tables E and F.

Irrespective of ACEs, females report significantly higher levels of poor mental health (anxiety: OR 2·4 [95% CI 1·84–3·01, *p* = 0·000]; somatization: OR 2·2 [95% CI 1·72–2·76, *p* = 0·000]; social dysfunction: OR 1·5 [95% CI 1·22–1·88, *p* = 0·000]; depression: OR 2·8 [95% CI 1·99–3·89, *p* = 0·000]; total GHQ: OR 2·6 [95% CI 2·02–3·43, *p* = 0·000]). No other socio-demographic variables included here significantly account for variations in mental health outcomes; apart from in the social dysfunction subscale, where *only* socio-demographic variables contribute to increased risk for greater social dysfunction.

A number of individual ACEs are associated with an increased risk for psychological distress. Retrospective emotional abuse/neglect is associated with increased anxiety (OR 1·8 [95% CI 1·32–2·36, *p* = 0·000]), depression (OR 1·6 [95% CI 1·08–2·25, *p* = 0·018]) and psychological distress in general (OR 1·6 [95% CI 1·18–2·17, *p* = 0·002]), while prospective emotional abuse/neglect is linked to increased psychological distress (OR 1·3 [95% CI 1·04–1·63, *p* = 0·034]). Retrospective exposure to severe household illness/disability is associated with increased somatization (OR 1·5 [95% CI 1·14–1·92, *p* = 0·004]); and retrospective exposure to IPV is associated with increased somatization (OR 1·7 [95% CI 1·19–2·46, *p* = 0·002]) and anxiety (OR 1·6 [95% CI 1·12–2·37, *p* = 0·010]). Both prospective physical and sexual abuse are associated with an increased risk of depression (OR 1·7 [95% CI 1·37–1·93, *p* = 0·034], and OR 1·8 [95% CI 1·27–2·07, *p* = 0·018], respectively). Prospective chronic household unemployment is associated with a decreased risk of somatization (OR 0·7 [95% CI 0·50–·99, *p* = 0·048]) and anxiety (OR 0·6 [95% CI 0·45–·91, *p* = 0·013]).

When looking at cumulative ACEs retrospectively, the greater the number of reported ACEs, the greater the risk for anxiety (OR 1·9 [95% CI 1·27–2·83, *p* = 0·002]), somatization (OR 1·5 [95% CI 1·14–2·06, *p* = 0·004]), depression (OR 1·6 [95% CI 1·07–2·31, *p* = 0·021]), and overall psychological distress (OR 1·9 [95% CI 1·26–2·99, *p* = 0·005]). Prospectively reporting four or more ACEs is associated with a greater than twofold increase in risk for somatization and overall psychological distress (OR 2·7 [95% CI 1·42–3·53, *p* = 0·003], and OR 2·2[95% CI 1·58–3·12, *p* = 0·008], respectively).

### 3.4 The influence of recent stressors

Reports of recent stressors show a strong and graded influence on anxiety, somatization, depression and overall psychological distress. The higher the number of recent stressors reported the greater the risk for negative mental health outcomes; the odds of experiencing somatization, anxiety and overall psychological distress more than doubling when four or more recent stressors are reported (OR 2·3 [95% CI 1·47–3·47, *p* = 0·000], OR 2·5 [95% CI 1·50–4·12, *p* = 0·000], and OR 2·5 [95% CI 1·63–3·96, *p* = 0·000], respectively). Recent stressors have a slightly weaker influence on depression but still increase the odds (OR 1.8 [95% CI 1·05–3·13, *p* = 0·034]). When accounting for recent stressors, adjusted odds ratios between retrospective ACEs and mental health outcomes decrease, suggesting that recent stressors independently contribute to poor mental health outcomes, but may well bias retrospective recall itself.

Factorial analysis of variance examining the effects of recent stressors and ACEs on psychological distress (Supplementary Table D) yielded significant models for both prospective ACEs [*F*(9, 1505) = 8·35, *p* *=* 0·000)] and retrospective ACEs [*F*(9, 1505) = 10·11, *p* *=* 0·000)]. For the prospective model, recent stressors (*p* = 0·000) and prospective ACEs (*p* *=* 0·023) were independently and significantly associated with psychological distress, but there was no significant interaction between recent stressors and prospective ACEs (*p* *=* 0·501). For the retrospective model, recent stressors (*p* = 0·000) and retrospective ACEs (*p* *=* 0·000) were independently and significantly associated with psychological distress, but again there was no significant interaction between recent stressors and retrospective ACEs (*p* *=* 0·976).

**Fig. 6:**
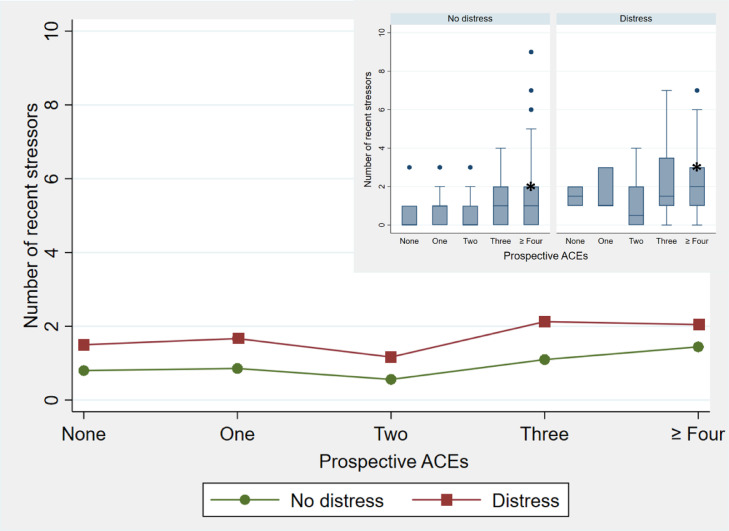
Comparison of the number of recent stressors by psychological distress for prospectively reported ACEs This figure shows in the larger graph the average number of recent stressors reported by participants presenting no distress on the GHQ (green, circle symbol) compared to those presenting with distress (maroon, square symbol) for categories of prospectively reported ACEs, with an inlay of the boxplot distribution of the same showing the median number of recent stressors, by distress status, as well as outliers. The boxes represent the interquartile range, with the median number of recent stressors indicated by the center line, and the whiskers represent the lowest and highest observations. No whiskers are visible where the lowest quartile is equal to the lowest observation or the upper quartile is equal to the highest.

**Fig. 7:**
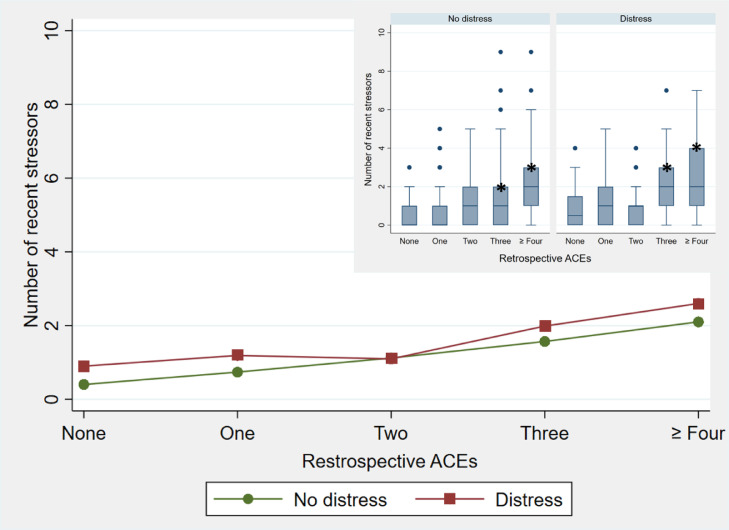
Comparison of the number of recent stressors by psychological distress for retrospectively reported ACEs This figure shows in the larger graph the average number of recent stressors reported by participants presenting no distress on the GHQ (green, circle symbol) compared to those presenting with distress (maroon, square symbol) for categories of retrospectively reported ACEs, with an inlay of the boxplot distribution of the same showing the number of recent stressors, by distress status, as well as outliers. The boxes represent the interquartile range, with the median number of recent stressors indicated by the center line, and the whiskers represent the lowest and highest observations. No whiskers are visible where the lowest quartile is equal to the lowest observation or the upper quartile is equal to the highest.

Fig. 6, Fig. 7 show the plotted analysis of variance results for the number of recent stressors reported in adulthood compared to the number of prospectively or retrospectively reported ACEs by psychological distress. The number of recent stressors reported in young adulthood appears to have less of an association with the number of ACEs prospectively experienced (Fig. 6) compared to the association between the number of recent stressors and retrospective ACEs (Fig. 7) where a steeper climb is apparent. For both retrospective and prospective ACEs, participants presenting with distress report on average more recent stressors than those not presenting with distress as the number of ACEs increases. The inlaid boxplots in Figs. 6 and 7 show the variability in recent stressors across prospective and retrospective ACEs, with less variance in the number of recent stressors when participants report lower numbers of ACEs compared to when they report *three* or *four or more* ACEs. For prospective ACEs the upper 25% of participants presenting with distress report on average higher numbers of recent stressors than their counterparts in each of the ACE categories. The same is true for retrospective ACEs apart from a smaller group of participants who present with distress, report relatively low ACEs (*two)*, and between zero and two recent stressors.

## 4 Discussion

The number of ACEs reported does not appear to have any association with social dysfunction. Instead, sociodemographic variables such as sex, socio-economic status and maternal education account for the variations in the social dysfunction scale. Given the socio-political climate in South Africa, characterized by a poor education system ‒ where about 60% of youth have either left school before graduation, or do not graduate [33], high youth unemployment rates, and resultant poverty, young adults may find themselves generally unprepared for functioning independently. This, and cultural issues specific to a young adult South African sample, could explain the contrasting findings on the social dysfunction subscale compared to the other subscales.

Extended to the other domains of the GHQ, the findings of this study indicate that both prospective and retrospective reports of ACEs can be linked to mental health in young adulthood. Young adults prospectively reporting four or more ACEs are more than twice as likely to experience psychological distress than those reporting less than four ACEs. While lower in prevalence than prospectively reported ACEs, retrospectively reported ACEs have a stronger association with anxiety, depression, somatization and general psychological distress in young adulthood. Similar studies have linked ACEs, individually and in combination, to mental health outcomes in general [26,34] and the GHQ in particular [35]. Heim and colleagues propose that early adverse experiences not only contribute to the manifestation of some types of depression, as evidenced in this study, but likely influence treatment responses [36].

Distal ACEs like chronic household unemployment, household legal trouble and low socio-economic status do not appear to have persistent influence over the mental health of young adults in this sample. Research has shown that relative to a history of either *no* or *high* cumulative lifetime adversity, a history of *some* adversity is associated with better mental health and wellbeing [37]. Counterintuitively, prospective chronic unemployment in the household appears to have a protective effect on somatization and anxiety in this study. The pervasiveness of adversity – in the distal form – throughout a community may in some sense lead to a ‘normalization’ of poverty and hardship that engenders a resilience in South African youth, mitigating its impact on mental health. In contrast, proximal ACEs such as physical, sexual and emotional abuse have a lasting effect on children, both in memory and in their effect on mental health, with retrospective and prospective physical abuse and emotional abuse/neglect and prospective sexual abuse linked to increased risk for depression, anxiety and general psychological distress.

The decrease in prevalence of retrospectively reported ACEs, compared to those reported prospectively, may be a result of a life view composed over time and filtered through recent stressors. Stressful life events are thought to negatively impact multiple areas of psychosocial functioning in general [38]. This study finds that there is an association between the number of recent stressors reported and the number of prospectively and retrospectively reported ACEs, that these reports of ACEs will be associated with poor mental health; but also that these associations are not straightforward. Young adults who prospectively report four or more ACEs, and those who retrospectively report any number of ACEs, with the strongest effect on four or more, are more likely to report a greater number of recent stressors. This effect of recent stressors has been assessed previously. One study found that stressful live events were associated with higher alcohol consumption among women exposed to childhood maltreatment, but could not find evidence for the role of recent events in the alcohol consumption of women not exposed to maltreatment [39]. Adversity experienced in childhood may sensitize individuals to future negative events, aggravating negative outcomes, which could explain the link between retrospective reporting, recent stressors and poorer later life outcomes. This finding may support a stress-sensitization hypothesis that early adversity leads to psychobiological changes that heighten sensitivity to subsequent stressors, altering strategies for coping with stress [40] and vulnerability to negative outcomes [41], [42], [43]. Harkness and colleagues assessed the relationship between childhood abuse and neglect and stressful life events for adolescents with depression and proposed that maltreatment may be an important risk factor that sensitizes individuals to the effects of acute independent life events [44]. Similar results were found in a psychiatric sample of adolescents; the timing and number of negative life events increased the risk of emotional and behavioral disorders by 3–6 times [45]. Honkalampi and colleagues suggest that ACEs may predispose individuals to depression, but current stressful events actualize these symptoms [46]. Recent stressors seem to reinforce a stress accumulation model — whereby early life stressors and subsequent stressors have unique and additive contributions; [47,48] supporting the idea that a number of mechanisms may work to link ACEs differentially to outcomes, whether psychological, physiological or behavioral. We propose that recent stressors have a confounding effort on the relationship between reports of ACEs and mental health outcomes; directly impacting mental health and possibly influencing autobiographical memory involved in retrospective recall. Further research should focus on the trajectories and pathways of groups of individuals who report prospective and retrospective ACEs and recent stressors.

While this study's findings show that males and females generally report similar numbers of ACEs prospectively, and males report significantly more ACEs retrospectively, females are up to twice as likely to suffer psychological distress as a result of these experiences. Stress-related disorders such as anxiety and depression are disproportionately prevalent in women. Literature suggests that stress and gonadal hormones may interact to predispose women to depression and anxiety; [49] adding further complexity in the form of sex to the relationship between ACEs, recent stressors and mental health. Mental health assessments that capture externalized emotions and behavior such as conduct disorder and aggression may better explain how adversity in childhood affects the mental health of young men.

Further research is required to tease out the mechanisms by which ACEs affect mental health using both subjective and objective, and prospective and retrospective reports. The continually contentious challenges around retrospective recall are somewhat allayed with emerging evidence of their links to both subjectively and objectively assessed negative outcomes. However, research cautions that psychopathology prior to or at the time of recall may bias memory towards either recalling a greater number of negative events, an exaggeration of some memories or a focus on painful ones [50]. This may denote a circular relationship between mental health and retrospective recall of ACEs or as Hardt and Rutter infer, that mental health is the filtering out of negative memories or their reimagining as benign [50].

The authors acknowledge a number of limitations in the study. The first set relate to the ACEs measure and method of collection. Measurement error in retrospective designs can be caused by a number of factors, including memory lapse over time [51], cognitive functioning at the time of the event in question [52], the non-disclosure – whether voluntary or involuntary ‒ of memories and specific details in the case of sensitive or traumatic experiences [51], and differential recall bias due to the participant's current life status and mood state at the time of reporting on past events [53]. Studies have cautioned on the sole use of retrospective accounts, particularly when assessing experiences prone to subjective judgment, but maintain that their usefulness in research cannot be negated and is enhanced when paired with additional sources, as in the current study [14,50,54]. With regard to the ACEs measure, the original ACEs inventory is limited in the type of adversities included which have been extended in this study and others, but room remains for further refinement of a more comprehensive set of ACEs. Additional limitations of ACE scores are that they do not distinguish between single episode, recurrent or chronic adversities and that the cumulative risk approach to scoring and summing of individual ACEs considers each as equivalent to the next. As a trade-off, the cumulative ACE score enables more precise and quantifiable examination and a number of studies have demonstrated a strong dose-response relationship between the ACE score and later life health and well-being outcomes [8,55,56].

The second set of limitations to this study relate to the data within Bt20+. At the 22–23-year data collection wave, and similar to all birth cohort studies, Bt20+ had an attrition rate of 50% due to a range of factors, including local migration patterns, which have been explored and described elsewhere [29,57]. Previous analysis on the same 22–23-year data on ACEs found no significant differences by sex and SES, the two covariates used in this study, between the participants surveyed and those not surveyed at the 22–23-year wave [58]. The authors make no claims about the generalizability of the results to the South African population but maintain that the initial cohort size (3273) was large enough at the start to mitigate the effects of attrition on internal validity.

In conclusion, this study finds the number, type and time of recall of ACEs have differential impacts on mental health in early adulthood. Both prospective and retrospective reports of ACEs are linked to psychological distress, with a stronger association between retrospective reports, and recent stressors reinforcing this relationship. Females are twice as likely to report poor mental health outcomes albeit reporting similar or fewer ACEs but the mental health assessment used in the study may not be capturing expressions of psychological distress in young males. The overall findings suggest that retrospective reports of ACEs can effectively screen for psychological distress in early adulthood and that the presence of recent stressors will be an exacerbating factor.

## Author contributions

SAN and LMR managed the study, including funding acquisitions, and oversaw implementation. SNN, SAN and LMR conceptualized the paper, curated and validated the data. SNN analysed the data. SNN and LMR wrote the manuscript with contributions from SAN. All authors reviewed the study findings and read and approved the final version before submission.

## Declaration of Competing Interest

The authors declare no competing interests.

## Appendix Supplementary materials

Image, application 1
