# Supplementary material for: Secondary analysis of retrospective and prospective reports of adverse childhood experiences and mental health in young adulthood: Filtered through recent stressors
Source: eClinicalMedicine. 2021 Aug 19;40:101094. doi: 10.1016/j.eclinm.2021.101094 (PMC8548929; doi:10.1016/j.eclinm.2021.101094)
Supplement: Supplementary file 1 [file mmc1.docx]

**Supplementary Materials**

**Table A. Adverse childhood experiences and recent stressors survey questions**

| **Adverse childhood experiences: Thinking back to your childhood, the first 18 years of your life, please tick each one that applies to you** | | | |
| --- | --- | --- | --- |
| **ACE** | **Question** | **Yes** | **No** |
| **Physical abuse** | Did a parent or other adult in the household **often or very often**…  Push, grab, slap, or throw something at you?  **OR**  **Ever** hit you so hard that you had marks or were injured? |  |  |
| **Sexual abuse** | Did an adult or person at least 5 years older than you **ever**…  Touch or fondle you or have you touch their body in a sexual way?  **OR**  Attempt or actually have oral, anal, or vaginal intercourse with you? |  |  |
| **Emotional abuse** | Did a parent or other adult in the household **often or very often**…  Swear at you, insult you, put you down, or humiliate you?  **OR**  Act in a way that made you afraid that you might be physically hurt? |  |  |
| **Emotional neglect** | Did you **often or very often** feel that …  No one in your family loved you or thought you were important or special?  **OR**  Your family didn’t look out for each other, feel close to each other, or support each other? |  |  |
| **Physical neglect** | Did you **often or very often** feel that …  You didn’t have enough to eat, had to wear dirty clothes, and had no one to protect you?  **OR**  Your parents were too drunk or high to take care of you or take you to the doctor if you needed it? |  |  |
| **Parental divorce** | Were your parents **ever** separated or divorced? |  |  |
| **Witnessing domestic violence** | Was your mother or stepmother:  **Often or very often** pushed, grabbed, slapped, or had something thrown at her?  **OR**  **Sometimes, often, or very often** kicked, bitten, hit with a fist, or hit with something hard?  **OR**  **Ever** repeatedly hit at least a few minutes or threatened with a gun or knife? |  |  |
| **Alcohol and/or drug abuse in the household** | Did you live with anyone who was a problem drinker or alcoholic or who used street drugs? |  |  |
| **Mental illness in the household** | Was a household member depressed or mentally ill, or did a household member attempt suicide? |  |  |
| **Legal trouble in the household** | Did a household member go to prison? |  |  |
| **Chronic illness (other than mental illness) in the household** | Was there anyone in your household who was chronically ill when you were a child? |  |  |
| **Unemployment of parent/caregiver** | Was one or more of your parents/caregivers mostly unemployed during your childhood because they could not get a job? |  |  |
| **Death of parent/caregiver** | Did either of your parents/caregivers pass away before you turned 18? |  |  |
| **Recent stressors: Please tick all appropriate events that have happened to you within the past 6 months** | | | |
| **Question** | | **Yes** | **No** |
| 1. During the last 6 months, has any household member (including you) been injured as a result of violence in the areas where you live or work? | |  |  |
| 1. During the last 6 months, has any household member (including you) been a victim of a violent crime (e.g. murder, robbery, mugging, assault, rape)? | |  |  |
| 1. During the last 6 months, did you witness a violent crime (e.g. murder, robbery, mugging, assault, rape)? | |  |  |
| 1. During the last 6 months, have you or anyone in your close family been seriously ill? | |  |  |
| 1. During the last 6 months, did any member of your close family pass away? | |  |  |
| 1. Is there anyone in your close family (including you) with a serious disability (for example, epilepsy, mental retardation, deafness, blindness, mental illness)? | |  |  |
| 1. Is there anyone in your close family (including you) that has a problem with drugs or alcohol? | |  |  |
| 1. During the last 6 months, have you had any serious fight or alienation from members of your family or your close neighbours? | |  |  |
| 1. During the last 6 months, have you or any member of your close family been arrested, had to go to court, or consulted a lawyer on a non-routine matter? | |  |  |

**Table B.** **Missing data on variables used in the study**

| Variable | No. Valid observations | No. missing observations | % missing observations |
| --- | --- | --- | --- |
| Exposures |  |  |  |
| Prospective ACE report | 1592 | 0 | 0 |
| Retrospective ACE report | 1592 | 0 | 0 |
| **Outcomes** |  |  |  |
| Somatization | 1574 | 18 | 1·13 |
| Anxiety | 1574 | 18 | 1·13 |
| Social dysfunction | 1575 | 17 | 1·07 |
| Depression | 1574 | 18 | 1·13 |
| GHQ total | 1536 | 56 | 3·52 |
| **Covariates** |  |  |  |
| Sex | 1589 | 3 | 0·19 |
| Ethnicity | 1589 | 3 | 0·19 |
| Socio-economic status | 1466 | 126 | 7·91 |
| Maternal education | 1476 | 116 | 7·29 |
| Negative recent life events | 1558 | 34 | 2·14 |
| ACE scores were computed for each participant who had data for at least 10 of the 13 ACEs; those with fewer than 10 – or 3 or more missing data points – were excluded from the analytic dataset. For prospective data, missing data was imputed from previous and subsequent waves of data to compose comprehensive accounts. The analysis was restricted to cases with data on the exposures, only cases with data for both prospective and retrospective ACEs were included. | | | |

**Table C: Comparison of the distribution of prospective and retrospective ACEs between cases with data and those without for variables included in the study**

| Variable | Prospective ACEs | | | | | | Retrospective ACEs | | | | | |
| --- | --- | --- | --- | --- | --- | --- | --- | --- | --- | --- | --- | --- |
|  | None | One | Two | Three | ≥ Four | p value | None | One | Two | Three | ≥ Four | p value |
| **Outcomes** |  |  |  |  |  |  |  |  |  |  |  |  |
| Somatization |  |  |  |  |  | 0·59 |  |  |  |  |  | 0·00 |
| No distress | 6 (0·6) | 17 (1·7) | 23 (2·3) | 79 (7·8) | 887 (87·6) |  | 99 (9·8) | 208 (20·6) | 180 (17·8) | 171 (16·9) | 354 (35·0) |  |
| Distress | 2 (0·4) | 7 (1·3) | 22 (3·9) | 40 (7·1) | 491 (87·4) |  | 31 (5·5) | 64 (11·4) | 111 (19·8) | 108 (19·2) | 248 (44·1) |  |
| Missing | 0 (0·0) | 2 (11·0) | 1 (5·6) | 1 (5·6) | 14 (77·8) |  | 9 (50·0) | 5 (27·8) | 2 (11·1) | 0 (0·0) | 2 (11·1) |  |
| Anxiety |  |  |  |  |  | 0·02 |  |  |  |  |  | 0·00 |
| No distress | 3 (0·3) | 17 (1·8) | 20 (2·1) | 73 (7·7) | 831 (88·0) |  | 95 (10·1) | 205 (21·7) | 193 (19·4) | 167 (17·7) | 294 (31·1) |  |
| Distress | 5 (0·8) | 7 (1·1) | 25 (4·0) | 46 (7·3) | 547 (86·8) |  | 35 (5·6) | 67 (10·6) | 108 (17·1) | 112 (17·8) | 308 (48·9) |  |
| Missing | 0 (0·0) | 2 (11·1) | 1 (5·6) | 1 (5·6) | 14 (77·8) |  | 9 (50·0) | 5 (27·8) | 2 (11·1) | 0 (0·0) | 2 (11·1) |  |
| Social dysfunction |  |  |  |  |  | 0·06 |  |  |  |  |  | 0·00 |
| No distress | 3 (0·5) | 10 (1·5) | 14 (2·1) | 55 (8·4) | 576 (87·5) |  | 57 (8·7) | 127 (19·3) | 106 (16·1) | 120 (16·2) | 248 (37·7) |  |
| Distress | 5 (0·6) | 14 (1·5) | 31 (3·4) | 64 (7·0) | 803 (87·6) |  | 73 (8·0) | 145 (15·8) | 185 (20·1) | 159 (17·3) | 355 (38·7) |  |
| Missing | 0 (0·0) | 2 (11·8) | 1 (5·9) | 1 (5·9) | 13 (76·5) |  | 9 (52·9) | 5 (29·4) | 2 (11·8) | 0 (0·0) | 1 (5·9) |  |
| Depression |  |  |  |  |  | 0·02 |  |  |  |  |  | 0·00 |
| No distress | 7 (0·5) | 21 (1·6) | 36 (2·7) | 110 (8·3) | 1160 (87·0) |  | 119 (8·9) | 247 (18·5) | 257 (19·3) | 242 (19·1) | 469 (35·2) |  |
| Distress | 1 (0·4) | 3 (1·3) | 9 (3·8) | 9 (3·8) | 218 (90·8) |  | 11 (4·6) | 25 (10·4) | 34 (14·2) | 37 (15·4) | 133 (55·4) |  |
| Missing | 0 (0·0) | 2 (11·1) | 1 (5·6) | 1 (5·6) | 14 (77·8) |  | 9 (50·0) | 5 (27·8) | 2 (11·1) | 0 (0·0) | 2 (11·1) |  |
| GHQ total |  |  |  |  |  | 0·14 |  |  |  |  |  | 0·00 |
| No distress | 5 (0·4) | 21 (1·9) | 27 (2·4) | 92 (8·2) | 983 (87·2) |  | 110 (9·8) | 232 (20·6) | 211 (18·7) | 202 (17·9) | 373 (33·1) |  |
| Distress | 2 (0·5) | 3 (0·7) | 18 (4·4) | 24 (5·9) | 361 (85·7) |  | 20 (4·9) | 37 (9·1) | 74 (18·1) | 72 (17·9) | 205 (50·3) |  |
| Missing | 1 (1·8) | 2 (3·6) | 1 (1·8) | 4 (7·1) | 48 (85·7) |  | 9 (16·1) | 8 (14·3) | 8 (14·3) | 5 (8·9) | 26 (46·3) |  |
| **Covariates** |  |  |  |  |  |  |  |  |  |  |  |  |
| Sex |  |  |  |  |  | 0·00 |  |  |  |  |  | 0·01 |
| Male | 3 (0·4) | 12 (1·6) | 13 (1·7) | 55 (7·2) | 681 (89·1) |  | 61 (8·0) | 132 (17·3) | 113 (14·8) | 138 (18·1) | 320 (41·9) |  |
| Female | 5 (0·6) | 14 (1·7) | 31 (3·76) | 65 (7·9) | 710 (86·1) |  | 78 (9·5) | 145 (17·6) | 179 (21·7) | 140 (17·0) | 283 (34·3) |  |
| Missing | 0 (0·0) | 0 (0·0) | 2 (66·7) | 0 (0·0) | 1 (33·3) |  | 0 (0·0) | 0 (0·0) | 1 (33·3) | 1 (33·3) | 1 (33·3) |  |
| Ethnicity |  |  |  |  |  | 0·00 |  |  |  |  |  | 0·76 |
| White | 0 (0·0) | 0 (0·0) | 0 (0·0) | 0 (0·0) | 1 (100·0) |  | 0 (0·0) | 0 (0·0) | 1 (100·0) | 0 (0·0) | 0 (0·0) |  |
| Black | 5 (0·4) | 24 (1·7) | 39 (2·7) | 107 (7·4) | 1266 (87·9) |  | 124 (8·6) | 248 (17·2) | 262 (18·2) | 257 (17·8) | 550 (38·2) |  |
| Mixed | 2 (1·5) | 2 (1·5) | 3 (2·2) | 11 (8·2) | 116 (86·6) |  | 12 (9·0) | 27 (20·2) | 26 (19·4) | 19 (14·2) | 50 (37·3) |  |
| Indian | 1 (7·69) | 0 (0·0) | 2 (15·4) | 2 (15·4) | 8 (61·5) |  | 3 (23·1) | 2 (15·4) | 3 (23·1) | 2 (15·4) | 3 (23·1) |  |
| Missing | 0 (0·0) | 0 (0·0) | 2 (66·7) | 0 (0·0) | 1 (33·3) |  | 0 (0·0) | 0 (0·0) | 1 (33·3) | 1 (33·3) | 1 (33·3) |  |
| Socio-economic status |  |  |  |  |  | 0·15 |  |  |  |  |  |  |
| Quintile 1 | 0 (0·0) | 4 (1·8) | 6 (2·7) | 20 (8·9) | 195 (86·7) |  | 14 (6·2) | 37 (16·4) | 31 (13·8) | 41 (18·2) | 102 (45·3) | 0·08 |
| Quintile 2 | 2 (0·7) | 5 (1·9) | 5 (1·9) | 14 (5·2) | 243 (90·3) |  | 23 (8·6) | 39 (15·5) | 53 (19·7) | 48 (17·8) | 106 (39·4) |  |
| Quintile 3 | 1 (0·2) | 3 (0·6) | 17 (3·3) | 38 (7·4) | 452 (88·5) |  | 39 (7·6) | 93 (18·2) | 95 (18·6) | 89 (17·4) | 195 (38·2) |  |
| Quintile 4 | 3 (1·0) | 6 (2·0) | 5 (1·7) | 22 (7·4) | 261 (87·8) |  | 32 (10·8) | 56 (18·9) | 62 (20·9) | 43 (14·5) | 104 (35·0) |  |
| Quintile 5 | 1 (0·6) | 4 (2·4) | 6 (3·7) | 20 (12·2) | 133 (81·1) |  | 18 (11·0) | 33 (20·1) | 34 (20·7) | 37 (22·6) | 42 (25·6) |  |
| Missing | 1 (0·8) | 4 (3·2) | 7 (5·6) | 6 (4·8) | 108 (85·7) |  | 13 (10·3) | 19 (15·1) | 18 (14·3) | 21 (16·7) | 55 (43·7) |  |
| Maternal education |  |  |  |  |  | 0·02 |  |  |  |  |  | 0·02 |
| No formal | 0 (0·0) | 2 (1·0) | 3 (1·6) | 16 (8·3) | 172 (89·1) |  | 15 (7·8) | 37 (19·2) | 29 (15·0) | 38 (19·7) | 74 (38·3) |  |
| Primary | 0 (0·0) | 5 (0·7) | 19 (2·8) | 52 (7·6) | 611 (88·9) |  | 42 (6·1) | 111 (16·2) | 127 (18·5) | 118 (17·2) | 289 (42·1) |  |
| Secondary | 5 (1·0) | 16 (3·3) | 14 (2·9) | 32 (6·5) | 425 (86·4) |  | 55 (11·2) | 90 (18·3) | 103 (20·9) | 86 (17·5) | 158 (32·1) |  |
| Tertiary | 2 (1·9) | 2 (1·9) | 4 (3·9) | 11 (10·6) | 85 (81·7) |  | 15 (14·4) | 22 (21·2) | 47 (13·5) | 18 (17·3) | 35 (33·7) |  |
| Missing | 1 (0·9) | 1 (0·9) | 6 (5·2) | 9 (7·8) | 99 (85·3) |  | 12 (10·3) | 17 (14·7) | 20 (17·2) | 19 (16·4) | 48 (41·4) |  |
| Negative recent life events |  |  |  |  |  | 0·01 |  |  |  |  |  | 0·00 |
| None | 3 (0·6) | 10 (2·1) | 26 (5·6) | 40 (8·6) | 389 (83·1) |  | 84 (18·0) | 136 (29·1) | 96 (20·5) | 69 (14·7) | 83 (17·7) |  |
| One | 3 (0·7) | 8 (1·9) | 6 (1·5) | 40 (9·7) | 357 (86·2) |  | 30 (7·3) | 75 (18·1) | 110 (26·6) | 67 (16·2) | 132 (31·9) |  |
| Two | 1 (0·3) | 3 (1·6) | 3 (1·6) | 13 (6·8) | 170 (89·5) |  | 10 (3·3) | 35 (11·4) | 50 (16·3) | 71 (23·2) | 140 (45·8) |  |
| Three | 1 (0·5) | 3 (1·6) | 3 (1·6) | 13 (6·8) | 170 89·5) |  | 3 (1·6) | 12 (6·3) | 19 (10·0) | 44 (23·2) | 112 (59·0) |  |
| ≥ Four | 0 (0·0) | 0 (0·0) | 2 (1·1) | 10 (5·6) | 168 (93·3) |  | 1 (0·6) | 7 (3·9) | 15 (8·3) | 24 (13·3) | 133 (73·9) |  |
| Missing | 0 (0·0) | 1 (2·9) | 3 (8·8) | 1 (2·9) | 29 (85·3) |  | 11 (32·4) | 12 (35·3) | 3 (8·8) | 4 (11·8) | 4 (11·8) |  |
| Significant *p* values are reflective of the small number of missing cases in each cell, biasing the distribution across ACEs. | | | | | | | | | | | | |

**Table D. Factorial ANOVA examining effects of retrospective and prospective ACEs and recent stressors on psychological distress**

| Source | Sum of squares | *df* | | Mean Square | *F* | *p* | Eta^2^ |
| --- | --- | --- | --- | --- | --- | --- | --- |
| Prospective ACEs, recent stressors and prospective ACEs by recent stressors (N=1515, R^2 =^ 0·0475, Adj· R^2^ = 0·0418) | | | | | | | |
| Model | 11161·92 | 9 | | 1240·21 | 8·35 | ·0000 | ·0475 |
| Recent stressors | 9344·04 | 4 | | 2336·01 | 15·72 | ·0000 | ·0401 |
| Prospective ACEs | 777·59 | 1 | | 774·59 | 5·21 | ·0226 | ·0034 |
| Prospective ACEs x Recent stressors | 497·92 | 4 | | 124·48 | 0·84 | ·5012 | ·0022 |
| Residual | 223645·07 | 1505 | | 148·60 |  |  |  |
|  |  |  | |  |  |  |  |
| **Retrospective ACEs, recent stressors and retrospective ACEs by recent stressors**  **N=1515, R^2 =^ 0·0696, Adj· R^2^ = 0·0546** | | | | | | | |
| Model | 13388·86 | 9 | | 1487·65 | 10·11 | ·0000 | ·0570 |
| Recent stressors | 4149·19 | 4 | | 1037·29 | 7·05 | ·0000 | ·0183 |
| Retrospective ACEs | 3218·75 | 1 | | 3218·75 | 21·88 | ·0000 | ·0143 |
| Retrospective ACEs x Recent stressors | 69·32 | 4 | | 17·33 | 0·12 | ·9762 | ·0003 |
| Residual | 221418·12 | 1505 | |  |  |  |  |
|  | | |  | | | | |
| **Prospective ACES, retrospective ACEs and prospective by retrospective ACEs on somatization**  **N=1574, R^2 =^ 0·0135, Adj· R^2^ = 0·0117** | | | | | | | |
| Model | 334·67 | 3 | | 111·56 | 7·18 | ·0001 | ·0135 |
| Prospective ACEs | 16·80 | 1 | | 16·80 | 1·08 | ·2985 | ·0006 |
| Retrospective ACEs | 272·91 | 1 | | 272·91 | 17·57 | ·0000 | ·0110 |
| Prospective ACEs x Retrospective ACEs | 9·96 | 1 | | 9·96 | 0·64 | ·4234 | ·0004 |
| Residual | 24380·01 | 1570 | | 15·53 |  |  |  |
|  | | | | | | | |
| **Prospective ACES, retrospective ACEs and prospective by retrospective ACEs on anxiety**  **N=1574, R^2 =^ 0·0461, Adj· R^2^ = 0·0443** | | | | | | | |
| Model | 1864·94 | 3 | | 621·61 | 25·31 | ·0000 | ·4613 |
| Prospective ACEs | 10·25 | 1 | | 10·25 | 0·42 | ·5184 | ·0003 |
| Retrospective ACEs | 1717·10 | 1 | | 1717·10 | 69·91 | ·0000 | ·0426 |
| Prospective ACEs x Retrospective ACEs | 16·19 | 1 | | 16·19 | 0·66 | ·4168 | ·0004 |
| Residual | 38560·62 | 1570 | | 24·56 |  |  |  |
|  | | | | | | | |
| **Prospective ACES, retrospective ACEs and prospective by retrospective ACEs on social dysfunction**  **N=1575, R^2 =^ 0·0009, Adj· R^2^ = 0·0010** | | | | | | | |
| Model | 15·39 | 3 | | 5·13 | 0·45 | ·7160 | ·0008 |
| Prospective ACEs | 4·74 | 1 | | 4·75 | 0·42 | ·5180 | ·0002 |
| Retrospective ACEs | 5·09 | 1 | | 5·09 | 0·45 | ·5035 | ·0002 |
| Prospective ACEs x Retrospective ACEs | 1·29 | 1 | | 1·29 | 0·11 | ·7361 | ·0000 |
| Residual | 17842·40 | 1571 | | 11·36 |  |  |  |
|  | | | | | | | |
| **Prospective ACES, retrospective ACEs and prospective by retrospective ACEs on depression**  **N=1574, R^2 =^ 0·0475, Adj· R^2^ = 0·0457** | | | | | | | |
| Model | 1150·77 | 3 | | 383·59 | 26·13 | ·0000 | ·0475 |
| Prospective ACEs | 286·90 | 1 | | 286·90 | 19·54 | ·0000 | ·0122 |
| Retrospective ACEs | 630·87 | 1 | | 630·87 | 42·97 | ·0000 | ·0266 |
| Prospective ACEs x Retrospective ACEs | 59·65 | 1 | | 59·65 | 4·06 | ·0440 | ·0026 |
| Residual | 23050·76 | 1570 | | 14·68 |  |  |  |
|  | | | | | | | |
| **Prospective ACES, retrospective ACEs and prospective by retrospective ACEs on total GHQ**  **N=1536, R^2 =^ 0·0390, Adj· R^2^ = 0·0371** | | | | | | | |
| Model | 9236·22 | 3 | | 3078·74 | 20·73 | ·0000 | ·0390 |
| Prospective ACEs | 605·85 | 1 | | 605·85 | 4·08 | ·0436 | ·0027 |
| Retrospective ACEs | 7275·73 | 1 | | 7575·43 | 48·98 | ·0000 | ·0309 |
| Prospective ACEs x Retrospective ACEs | 209·03 | 1 | | 209·03 | 1·41 | ·2357 | ·0009 |
| Residual | 227569·22 | 1532 | | 148·54 |  |  |  |
| Factorial analysis of variance showing unique contribution of prospective and retrospective reports of ACEs, as well as any interactions between them, to the variance in each mental health outcome as well as for the interaction effects between retrospectively and prospectively reported ACEs and recent stressors on psychological distress. | | | | | | | |

**Tables E. Unadjusted effects of reported ACEs on mental health**

|  | **Somatization**  **OR [CI 95%]** | **Anxiety**  **OR [CI 95%]** | **Social dysfunction**  **OR [CI 95%]** | **Depression**  **OR [CI 95%]** | **GHQ Total**  **OR [CI 95%]** |
| --- | --- | --- | --- | --- | --- |
| **Prospective ACEs** |  |  |  |  |  |
| Physical abuse | 1·08 [·88-1·36] | 1·18 [·95-1·47] | ·89 [·72-1·11] | 1·53 [1·13-2·09]** | 1·23 [·96-1·58] |
| Sexual abuse | 1·22 [·98-1·52] | 1·06 [·86-1·32] | ·92 [·74-1·14] | 1·67 [1·25-2·24]** | 1·34 [1·05-1·71]* |
| Emotional abuse/neglect | 1·29 [1·04-1·62]* | 1·32 [1·07-1·65]* | 1·13 [·91-1·41] | 1·17 [·87 1·57] | 1·37 [1·08-1·76]** |
| Child separation | 1·29 [·96-1·71] | 1·18 [·88-1·57] | 1·24 [·93-1·67] | 1·04 [·71-1·55] | 1·27 [·93-1·74] |
| Divorce/separation | ·89 [·72-1·11] | ·94 [·77-1·17] | ·99 [·80-1·21] | ·99 [·75-1·33] | ·89 [·70-1·13] |
| Parental death | ·88 [·68-1·13] | ·94 [·73-1·21] | 1·12 [·87-1·44] | 1·23 [·89-1·71] | 1·10 [·83-1·45] |
| Exposure to violence | 1·08 [·84-1·41] | ·81 [·63-1·04] | 1·01 [·78-1·30] | 1·04 [·72-1·49] | 1·00 [·75-1·33] |
| Exposure to IPV | ·88 [·69-1·12] | 1·15 [·91-1·46] | ·93 [·73-1·17] | ·99 [·72-1·37] | ·91 [·70-1·19] |
| Chronic unemployment | ·69 [·49-·98]* | ·70 [·50-·96]* | ·66 [·47-·93]* | ·73 [·47-1·14] | ·71 [·50-1·02] |
| Household substance abuse | ·97 [·78-1·21] | ·94 [·76-1·16] | 1·06 [·86-1·31] | 1·05 [·79-1·41] | ·99 [·78-1·26] |
| Household legal trouble | ·90 [·72-1·13] | ·93 [·74-1·16] | ·94 [·76-1·17] | 1·23 [·92-1·66] | ·99 [·78-1·27] |
| Household illness/disability | ·97 [·77-1·22] | ·95 [·76-1·16] | 1·14 [·92-1·43] | 1·05 [·77-1·44] | 1·02 [·79-1·32] |
| Household death | 1·04 [·83-1·31] | 1·07 [·86-1·34] | ·97 [·78-1·21] | 1·15 [·84-1·56] | 1·13 [·88-1·46] |
| **Pros ACE category** 0 (ref) |  |  |  |  |  |
| 1 | 1·24 [·19-7·67] | ·25 [·46-1·32] | ·84 [·16-4·35] | 1·01 [·09-11·24] | ·36 [·05-2·74] |
| 2 | 2·9 [·25-15·77] | ·75 [·16-3·52] | 1·33 [·28-6·35] | 1·75 [·19-16·09] | 1·67 [·29-9·54] |
| 3 | 1·51 [·29-7·87] | ·38 [·09-1·66] | ·69 [·16-3·05] | ·57 [·06-5·18] | ·65 [·12-3·57] |
| ≥4 | 1·67 [·34-8·29] | ·39 [·09-1·65] | ·84 [·20-3·54] | 1·3 [·16-10·86] | ·92 [·18-4·76] |
| **Binary Pros ACEs** Less than 4 (ref) |  |  |  |  |  |
| Four or more | 1·84 [·64-2·27]** | 1·08 [·88-1·32] | 1·05 [·86-1·29] | 1·69 [1·28-2·23]*** | 1·35 [1·07-1·69]* |
| **Total Pros ACE score** | 1·00 [·96-1·05] | ·99 [·96-1·04] | ·99 [·95-1·03] | 1·12 [1·06-1·19]*** | 1·05 [1·01-1·10]* |
| **Retrospective ACEs** |  |  |  |  |  |
| Physical abuse | ·89 [·52-·1·51] | 1·77 [1·05-3·00]* | ·91 [·55-1·52] | 1·43 [·77-2·67] | 1·7 [·67-2·07] |
| Sexual abuse | 1·56 [·81-3·02] | 2·15 [1·10-4·19]* | 1·49 [·77-2·91] | 1·98 [·90-4·34] | 1·88 [·93-3·79] |
| Emotional abuse/neglect | 1·10 [·79-1·52] | 1·28 [1·04-1·76]* | ·96 [·71-1·31] | 1·56 [1·02-2·41]* | 1·44 [1·01-2·05]* |
| Divorce/separation | ·86 [·65-1·15] | ·93 [·70-1·24] | ·77 [·59-1·00] | 1·39 [·94-2·07] | 1·10 [·80-1·51] |
| Parental death | 1·08 [·77-1·52] | ·90 [·64-1·27] | 1·03 [·75-1·43] | ·99 [·62-1·57] | ·95 [·65-1·39] |
| Exposure to violence | 1·27 [·93-1·74] | 1·38 [1·01-1·89]* | 1·26 [·93-1·71] | 1·17 [·77-1·79] | 1·37 [·97-1·94] |
| Exposure to IPV | 1·67 [1·12-2·51]* | 1·52 [1·01-2·03]* | 1·82 [1·19-2·76]** | ·84 [·49-1·46] | 1·49 [·97-2·32] |
| Chronic unemployment | 1·14 [·85-1·53] | 1·21 [·90-1·61] | ·79 [·59-1·05] | 1·22 [·81-1·83] | ·88 [·63-1·22] |
| Household substance abuse | 1·40 [·99-1·97] | 1·17 [·84-1·64] | 1·13 [·82-1·57] | 1·34 [·85-2·09] | 1·37 [·95-1·98] |
| Household legal trouble | ·78 [·54-1·10] | 1·46 [1·04-2·07]* | 1·10 [·78-1·55] | ·88 [·55-1·42] | ·84 [·56-1·24] |
| Household illness/disability | 1·6 [1·19-2·17]** | 1·57 [1·17-2·11]** | 1·00 [·75-1·34] | 1·75 [1·16-2·64]** | ·135 [·97-1·88] |
| Household death | ·89 [·66-1·21] | 1·22 [·91-1·65] | 1·09 [·82-1·45] | 1·31 [·87-1·98] | 1·34 [·97-1·87] |
| **Retro ACE category** 0 (ref) |  |  |  |  |  |
| 1 | ·97 [·60-1·55] | ·85 [·53-1·35] | ·85 [·57-1·29] | 1·03 [·51-2·07] | ·80 [·45-1·40] |
| 2 | 1·87 [1·19-2·94]** | 1·54 [·99-2·39] | 1·28 [·85-1·94] | 1·26 [·64-2·48] | 1·74 [1·03-2·93]* |
| 3 | 1·91 [1·21-3·02]** | 1·74 [1·12-2·72]* | ·97 [·64-1·47] | 1·46 [·75-2·84] | 1·77 [1·05-2·98]* |
| ≥4 | 2·12 [1·39-3·23]*** | 2·73 [1·82-4·10]*** | 1·05 [·72-1·53] | 2·70 [1·48-4·94]** | 2·72 [1·69-4·39]*** |
| **Binary Retro ACEs** Less than four |  |  |  |  |  |
| More than four | 1·47 [1·19-1·81]*** | 2·11 [1·72-2·6]*** | 1·04 [·85-1·28] | 2·29 [1·74-3·03]*** | 2·04 [1·62-2·57]*** |
| **Total Retro ACE score** | 1·15 [1·11-1·21]*** | 1·26 [1·19-1·33]*** | 1·00 [·95-1·05] | 1·21 [1·13-1·29]*** | 1·23 [1·16-1·29]*** |
| Logistic regression models fitted for each individual ACE, followed by each composite measure of ACEs, separately for prospective and retrospective ACEs.  * *p*<·05 ***p*<·01 ****p*<·0001 | | | | | |

**Table F. Adjusted effects of reported ACEs on mental health**

|  | **Somatization**  **OR [CI 95%]** | **Anxiety**  **OR [CI 95%]** | **Social dysfunction**  **OR [CI 95%]** | **Depression**  **OR [CI 95%]** | **GHQ Total**  **OR [CI 95%]** |
| --- | --- | --- | --- | --- | --- |
| **Sex** (Male ref) |  |  |  |  |  |
| Female | 2·18 [1·72-2·76]*** | 2·35 [1·84-3·01]*** | 1·51 [1·22-1·88]*** | 2·79 [1·99-3·89]*** | 2·63 [2·02-3·43]*** |
| **SES** (Quintile 1 ref) |  |  |  |  |  |
| Quintile 2 | 1·02 [·68-1·54] | ·84 [·55-1·28] | ·83 [·57-1·22] | 1·09 [·65-1·84] | 1·01 [·66-1·56] |
| Quintile 3 | 1·03 [·72-1·48] | ·88 [·61-1·28] | ·83 [·59-1·75] | 1·05 [·65-1·70] | ·85 [·57-1·26] |
| Quintile 4 | ·99 [·66-1·49] | ·87 [·58-1·32] | ·66 [·45-·97]* | 1·07 [·62-1·84] | ·97 [·63-1·59] |
| Quintile 5 | ·84 [·61-1·58] | ·98 [·64-1·67] | 1·03 [·66-1·62] | 1·01 [·53-1·93] | ·96 [·57-1·60] |
| **Maternal education** No schooling (ref) |  |  |  |  |  |
| Primary school | 1·14 [·78-1·66] | 1·09 [·74-1·60] | ·80 [·56-·98]* | ·96 [·60-1·54] | ·90 [·60-1·35] |
| Secondary school | 1·18 [·79-1·76] | 1·27 [·84-1·92] | ·99 [·69-1·45] | ·68 [·41-1·17] | 1·05 [·68-1·62] |
| Post-secondary | 1·59 [·92-2·77] | 1·39 [·79-2·46] | ·79 [·47-1·34] | ·85 [·39-1·82] | ·99 [·54-1·84] |
| **Recent stressors** 0 (ref) |  |  |  |  |  |
| 1 | 1·26 [·92-1·74] | 1·55 [1·11-2·17]* | 1·20 [·90-1·60] | ·97 [·61-1·54] | 1·02 [·71-1·47] |
| 2 | 1·54 [1·08-2·19]* | 1·72 [1·18-2·51]** | 1·43 [·99-1·96] | 1·38 [·85-2·22] | 1·71 [1·17-2·50]** |
| 3 | 2·09 [1·38-3·15]*** | 2·12 [1·36-3·30]** | 1·23 [·85-1·77] | 1·30 [·75-2·27] | 1·98 [1·28-3·07]** |
| ≥4 | 2·26 [1·47-3·47]*** | 2·45 [1·50-4·12]*** | 1·11 [·76-1·62] | 1·81 [1·05-3·13]* | 2·5 [1·63-3·96]*** |
| **Prospective ACEs** |  |  |  |  |  |
| Physical abuse |  |  |  | 1·67 [1·37-1·93]* |  |
| Sexual abuse |  |  |  | 1·79 [1·27-2·07]* | 1·20 [·92-1·58] |
| Emotional abuse/neglect | 1·17 [·91-1·48] | 1·14 [·89-1·46] |  |  | 1·25 [1·04-1·63]* |
| Chronic unemployment | ·70 [·50-·99]* | ·64 [·45-·91]* | ·88 [·63-1·23] |  |  |
| **Binary Prospective ACEs** (<4 ref) |  |  |  |  | 1·10 [·83-1·45] |
| ≥4 | 2·73 [1·42-3·53]** |  |  | 1·30 [·91-1·86] | 2·23 [1·58-3·12]** |
| **Retrospective ACEs** |  |  |  |  |  |
| Physical abuse |  | 1·83 [1·16-2·89]* |  |  |  |
| Sexual abuse |  | 1·47 [·81-2·65] |  |  |  |
| Emotional abuse/neglect |  | 1·76 [1·32-2·36]*** |  | 1·56 [1·08-2·25]* | 1·60 [1·18-2·17]** |
| Exposure to IPV | 1·71 [1·19-2·46]** | 1·63 [1·12-2·37]* | 1·29 [·92-1·82] |  |  |
| Exposure to violence |  | 1·13 [·82-1·55] |  |  |  |
| Household illness/disability | 1·48 [1·14-1·92]** | 1·14 [·87-1·49] |  | 1·19 [·84-1·69] |  |
| Household legal trouble |  | 1·32 [·97-1·79] |  |  |  |
| **Binary Retrospective ACEs** (<4 ref) |  |  |  |  |  |
| ≥4 | 1·51 [1·14-2·06]** | 1·91 [1·27-2·83]** |  | 1·55 [1·07-2·31]* | 1·94 [1·26-2·99]** |
| Adjusted logistic regression models with covariates sex, socioeconomic status, maternal education and recent stressors. Five separate models, one per outcome, are fitted with significant predictors from unadjusted models and include both prospective and retrospective ACEs; the ORs for prospective ACEs indicates the unique contribution of that predictor independently from retrospective ACEs and vice versa. Empty cells represent those predictors that were not entered into a model for that specific outcome.  * *p*<·05 ***p*<·01 ****p*<·0001 | | | | | |
